# Interaction between professionals and cancer survivors in the context of Brazilian and Canadian care ^1^

**DOI:** 10.1590/1518-8345.2253.2972

**Published:** 2017-12-21

**Authors:** Rafaela Azevedo Abrantes de Oliveira, Márcia Maria Fontão Zago, Sally Elizabeth Thorne

**Affiliations:** 2 Doctoral student, Escola de Enfermagem de Ribeirão Preto, Universidade de São Paulo, PAHO/WHO Collaborating Centre for Nursing Research Development, Ribeirão Preto, SP, Brazil. Scholarship holder at Fundação de Amparo à Pesquisa do Estado de São Paulo (FAPESP), Brazil.; 3 PhD, Professor, Escola de Enfermagem de Ribeirão Preto, Universidade de São Paulo, PAHO/WHO Collaborating Centre for Nursing Research Development, Ribeirão Preto, SP, Brazil.; 4 PhD, Professor, Nursing School, University of British Columbia, Vancouver, BC, Canada.

**Keywords:** Health Communication, Access to Information, Neoplasms, Survivors, Nursing Care, Qualitative Research

## Abstract

**Objective:**

analyze cancer survivors’ reports about their communication with health professional team members and describe the similarities and differences in interactional patterns between Brazilian and Canadian health care contexts.

**Method:**

This study adopted a qualitative health research approach to secondary analysis, using interpretive description as the methodology, allowing us to elaborate a new research question and look at the primary data from a different perspective. There were in total eighteen participants; all of them were adults and elderly diagnosed with urologic cancer. After being organized and read, the data sets were classified into categories, and an analytic process was performed through inductive thematic analysis.

**Results:**

This resulted in three categories of findings which we have framed as: Communication between professional and survivor; The symptoms, the doubts, the questions; and Actions and reaction.

**Conclusion:**

This comparative study allowed us to bring to the attention of health professionals, especially nurses, findings regarding effective communication, humanization and empathy, supporting both inside and outside support groups, giving pieces of advice, and advocating for the survivor as is necessary. The study also showed the importance of self-development of these professionals as they fight for better quality in the health system for their patients.

## Introduction

Cancer survivorship represents the state or process of living after the cancer diagnosis, living with cancer, through and beyond it. The term is used by healthcare professionals, researchers and patients themselves with cancer, extending to their families. It is a concept that seeks not only to identify and understand the survivors’ physical, social, spiritual and psychological issues, but also offers the idea that proper care involves promoting quality of life for this group^1^ ^-^ ^3^. Thus, in order to understand and address these questions, the cancer survivorship care plan acts as a tool that facilitates the coordination of care by the survivors themselves, the multidisciplinary team, and others involved^4^.

It is the time after the end of primary treatment that is neglected by health professionals and researchers though. At this moment, the survivors become confused and are confronted with new challenges and anxieties in returning to their “normal life”^5^. Following on this idea, professional and patient interaction should help the survivors rebuild a new normality. Nevertheless, as the authors^3^ delineated, there are still barriers that harm the quality of care in the cancer survivorship process. Among those that matter most is what the authors highlight as poor mechanisms for communication. Communication is complex, dynamic and linked to the context^6^, it is difficult to quantify and control^7^; furthermore, it is strongly influenced by behaviors of both the patient and the health professional^5^ ^,^ ^8^. The literature related to communication in the cancer context indicates that the patient and professional relationship is vital to survivors’ quality of life and a powerful factor in the quality of care^9^. Moreover, effective communication is fundamental and is directly linked to the patient’s well-being and satisfaction with care services^9^ ^-^ ^11^.

Therefore, in the context of a research internship in Vancouver, Canada, one of the authors had the opportunity to make a technical visit and meet members of the health system at an oncologic hospital in Vancouver, which is a reference cancer treatment center and also delivers follow-up care to cancer patients. Because of this opportunity, she realized that, in the context of her country’s health system (Brazil), there is still a lot to be done; there are operational barriers in the health system that need to be surpassed and new frontiers that have to be achieved. On the basis of this insight, in analyzing both the Canadian data and data collected during her doctoral research in Brazil, the researcher realized that the patients from both countries always - directly or indirectly - expressed at some point within their discourse either praise or criticism of the health care system and their relationship with the health professionals. Thus, recognizing that communication is a duty of nursing, the following research question was asked: How do urologic cancer survivors’ reports about their communications with health professional team members reveal similarities and differences in interactional patterns between the Brazilian and Canadian health care contexts?

## Aim

To analyze cancer survivors’ reports about their communication with health team members and describe the similarities and differences in interactional patterns between the Brazilian and Canadian health care contexts.

## Method

This study was a qualitative study with interpretive description as a methodology. Interpretive description is designed for the explicit logic of knowledge development in the applied health disciplines. Rather than grounding theoretical scaffolding in the social sciences from which conventional qualitative approaches emerged, this method draws upon disciplinary logic to guide the inquiry process toward knowledge forms with direct practice utility^9^ ^,^ ^12^.

Secondary analysis is a strategy that allows us to elaborate new research questions and look at the primary data from a new perspective; thus, it explores existing data in a manner that was not previously explored. Moreover, although connections between professional team members and patients’ interactions were not the focus of the original interviews, these themes were clearly represented in the data from both studies and largely informed the secondary research questions^13^. In other words, despite the fact that the Canadian data focused on participants’ experiences of helpful and unhelpful cancer communication, and the Brazilian data focused on cancer survivors’ experience after the treatment, the authors reviewed the data sets to address this new research question, related to the theme of the original studies but not formally examined^12^. Thus, the secondary analysis process created the potential for the development of wider theories about cancer communication through a comparison of two distinct and theoretically representative databases^14^. 

Because qualitative inquiries often involve intensive data collection using methods such as semi-structured interviews, participant observation and fieldwork approaches, they typically create data sets that contain a wealth of information beyond what can be included in a primary research report. Furthermore, as time goes by, new questions often arise for which existing data sets may be an efficient and appropriate source of grounded knowledge^13^. Qualitative secondary analysis is a method for study of phenomena within a data set that would not have been considered significant at the original time of inquiry. Moreover, it can be an efficient and effective way to pose questions that extend beyond the scope of any individual research team’s findings^13^.

The form of secondary analysis used here is “analytic expansion” - a term used to refer to the kind of study in which a researcher makes further use of a primary data set in order to ask new or emerging questions that derive from having conducted the original analysis but were not envisioned within the original scope of the primary studies aims^15^.

The primary dataset is from Brazil. It consists of nine Brazilian participants diagnosed with urological cancer who were interviewed. All of them had been followed at the Ribeirão Preto *Hospital das Clínicas*. The sample included adult patients and patients over 60 years of age (mean=59.2). The majority of them was married and possessed a low level of education (elementary school); only two participants had finished high school and one had not finished higher education. All of them were diagnosed with urological cancer (kidney, bladder, testicular and prostate) and / or under treatment at the Urologic Oncology Clinic of the University Hospital at Ribeirão Preto Faculty of Medicine, University of São Paulo (HCRP-FMRP / USP). Participants were recruited into the study regardless of educational or socio-economic level, or reported physical and psychological conditions. Individuals were considered eligible for participation if they had performed treatment for at least three months. 

The interviews had a minimum duration of one hour and a maximum of two hours. They were scheduled and recorded with the consent of the participants and took place according to the participants’ availability to receive the researcher at their residences. Several interviews were obtained from each participant until we were able to achieve the study objectives. During the meetings, we observed the environment, the contacts between family and friends, paying attention to the nonverbal messages displayed and participating in some daily activities.

The project was submitted to and approved by the Research Ethics Committee at the Ribeirão Preto College of Nursing from University of São Paulo (process 503.385, December 9th, 2013). The patients were assured of their rights to be informed about the study and freely consent to participate in it, also being assured of the confidentiality of identification and the information that was provided. 

The second dataset is from Canada. The data for the Canadian sample was part of a larger database consisting of interviews (an average of three for each participant) with nine men ranging in age from 54 to 81 (mean=69.2) and with primary prostate cancer. The participants included those diagnosed with early and advanced stages of cancer, some living with metastatic disease, and others who self-identified as cured or in remission. All participants self-identified as Caucasian (n=9). Regarding the educational level, while a few had high-school education, the majority of the participants had concluded university or college and most of them were married, while one was single. The characteristics of all Canadian and Brazilian participants are described in Table 1, as follows. 

**Table 1:** Sociodemographic information of Brazilian and Canadian participants. Ribeirao Preto, SP, Brazil, 2015 and Vancouver, BC, Canada, 2010

	**Brazil**	**Canada**
**Gender**		
**Male**	**9**	**9**
**Type of cancer**		
**Prostate**	**4**	**9**
**Kidney**	**3**	**-**
**Bladder**	**1**	**-**
**Testicle**	**1**	**-**
**Age**	**40-78 (mean= 59.2)**	**54-81 mean=69.2)**
**Education**		
**Elementary school**	**8**	**-**
**High school**	**-**	**5**
**Incomplete higher education**	**1**	**-**
**Higher education**	**-**	**4**
**Marital status**		
**Married**	**5**	**6**
**Widow**	**1**	**-**
**Single**	**-**	**1**
**Divorced**	**3**	**2**
**Race**		
**Caucasian**	**2**	**9**
**Black**	**6**	**-**
**Brown**	**1**	**-**

The original study using this subset of survivors^9^ used interpretive description^12^ and nursing’s disciplinary epistemology to frame the study and conduct the analysis. Semi-structured/open-ended face-to-face interviews were conducted in the original study in a naturalistic setting to explore communication pertaining to the end of the primary treatment phase. Participants in the original study were volunteers, purposefully sampled through multiple approaches, who lived in British Columbia, Canada. Initial and ongoing ethical approval for the Communication Cancer Care database included informed consent to use de-identified transcripts for other research purposes in the future, beyond the original study (protocol H09-0171), Behavioral Research Ethics Board, University of British Columbia, initial approval July 27, 2009. 

Data analysis in relation to this subset follows the constant comparative approach inherent in interpretive description, in which each case is considered in the context of all others and of the dataset as a whole. In keeping with the logical model of a clinical discipline such as nursing, we do not only report on what we observed, but also on what we understand it to mean in relation to the problem we are addressing^9^. 

Within the interpretive description, we used thematic analysis as a method for identifying, analyzing and reporting patterns within data. This kind of analysis consists of an interpretive process of organizing the data based on searching for sense according to the commonalities, relationships and differences between cases^16^. Moreover, the analysis is not a linear process where you simply move from one phase to the next. Instead, it is a more recursive process, where you move back and forth as needed, throughout the six phases of familiarization with the data, generating initial codes, searching for themes, reviewing themes, defining and naming themes, and producing the report^16^.

## Results

Through the data analysis process, it was observed that patients in both Brazilian and Canadian contexts have problems with communication and relationships within the professional-patient dyad. On the other hand, over time, we noticed some advances that might help improve this communication and, consequently, the care practice.

The first category is communication between professional and survivor. From the moment the patient starts to feel the symptoms that make him/her seek health services, effective communication needs to be established so the whole care process can be conducted in the best way possible and the patients feel they are in a trusting and warm atmosphere. The reality can be very different from theory though. Already at the diagnosis moment, a lot of patients criticize the doctor’s communication approach of the cancer diagnosis: *The doctor told me: You have cancer…You might live thirty years! I didn’t ask him how long I’m going to live and all this stuff* (C); *They [the doctors] could talk with more humanity! I don’t know how it should be told they have cancer or not, but they studied more than I did, they are supposed to know!* (B). In the first example, the Canadian patient complains about the way the doctor approached the diagnosis. The Brazilian follows the same pattern but transmits in his discourse that the diagnosis could have been told in a more humanized way and, despite claiming little knowledge, the doctors are instructed and should know how to proceed in the situation. It has to be taken into consideration that this moment of the diagnosis is crucial. Once the information related to something so very important and emotionally destabilizing is conveyed, it makes reference to a pathology that patients associate with the idea of death. Therefore, the manner in which the diagnosis is transmitted to the patient says a lot about the impressions he or she would have about the health professional. 

Another question that was addressed is the language the professionals use when they are establishing communication with the patient. A Canadian patient reported: *They have to realize they’re dealing with people who are pretty ignorant about the fact, so give it a bit of time and give it a bit of an explanation and make the person feel at ease, not just say bingo* (C). Similarly, the Brazilian went straight to the point: *They say what they have to say. I even ask, but I don’t understand. That’s why my daughter goes with me every time she can. She understands it better* (B). This is one of the barriers identified in the literature related to professional-patient interaction. The professionals need to recognize the listener, his or her context and culture, and try to use an adequate language.

In the Brazilian context, specifically in a city in the countryside of the state of São Paulo, the survivors perform the follow-up with urology oncologic doctors. In this context, the main question raised by the patients relates to the frequent rotation among the doctors. In the survivors’ opinion, they should be taken care of by a single doctor. *It would be way better if there was just one. I never got used to it, but what can I do?* (B). This frequent rotation generates a problematic understanding, absence of bonding and, although professionals speak the same language and share the same information, patients report they would like to be able to get closer and bond with just one professional: *I know there are many [doctors]. That’s bad. Sometimes I’m treated by a nice doctor who talks a lot, sometimes by one who’s more boring! I wish there was just one, that way we could become friends [laughs]* (B). It is important to highlight that this turnover is common in Brazilian teaching hospitals.

Surely, information is what patients want and, regardless of the country, all of them want to feel safe about what is happening to their bodies. As one explains, *It’s very important that I understand what’s going on in my body and communication is vital* (C). Another completes the thought, *Communication is about being sympathetic or being empathic* (C). Along the cancer trajectory, the patients become involved with their health care team and build more trustworthy links and relationships, where doubts, fears, and uncertainties that used to hover in their heads at the moment of the diagnosis tend to diminish with time and those groups of professionals become part of their support group during the whole process. 

The second category corresponds to the symptoms, the doubts, the questions, where to find information? The search for information starts with a doubt the patient is left with right after feeling an unexpected symptom *I think it’s very important looking for answers because most people want to know, you know like prostate, different kinds of cancers have different outcomes, long-term outcomes, potential outcomes and even within prostate cancer* (C). The point is where to take those doubts? *I went to the Internet and I read up everything I could find on prostate cancer* (B). Another said, *I searched on the Internet and I got scared*! (C). Both Brazilians and Canadians reported that they searched for information on the Internet, especially when they were anxious because of a certain doubt. In the Brazilian context, follow-up patients have to wait a considerable amount of time between appointments and, as a result, because they do not have patience to wait for the next appointment to get those doubts answered, they turn to the Internet in the end. How trustworthy the websites are that they locate, and how efficient they are for patients to use remains unknown though. 

In the Canadian context, due to the level of instruction and organization of the health services, the patients are willing to read books, brochures and to access recommended websites by the professionals or by the institution. *I have a lot of options! My doctor gave me a book about prostate cancer once and it was really good. Furthermore, we have excellent websites or nurses who can help us anytime* (C). This kind of attitude in which the professional helps patients in their search for information to construct knowledge about what is about to come in their lives is something that does not happen, or at least happens infrequently, in Brazil, chiefly because of work overload. Instead, in the context of Brazilian public health, there are vehicles that broadcast information, such as television and radio, that are misunderstood by the patient and are associated with a group of patients with lower educational levels: *I think cancer has cure because I saw on television [laughs], but I think it depends on the type of cancer, the patient* (B). 

In addition to a concern about facts, seeking information confronts patients with a series of issues; the great amount of time between the appointments, where to seek information, which sources are reliable and the work overload are problems that affect the interaction and communication, and, consequently, the care quality. The Brazilian issues of today were Canadian issues in the past and we see that, in the Canadian context, they have been partially or completely resolved. Both in Canada and in Brazil, there are websites created to give information to the patient; in Brazil, however, the divulging of information is still limited and does not cover the full range of patient concerns. 

Another point worth highlighting is the practicality of Canada’s telenursing system. To enhance the readers’ understanding, the patient care system in Canada is mediated by devices that help to overcome the barriers of distance and time. In Brazil, in 2007, the Ministry of Health implemented the Brazilian National Telehealth Network Program, with the aim of promoting Teleassistance and Teleducation to improve health care quality and basic health information in the Brazilian Unified Health System (SUS). Nevertheless, it is not possible yet to see the effects caused by the implementation of this program. Many professionals and patients do not even know about its existence. On the other hand, it seems a valid approach and deserves attention, since it allows nurses to use technology to assess, consult and give advice to the patient; to support the patient; to strengthen the patient; to teach the patient, and to facilitate the patient’s learning^17^.

The third category is actions and reaction: how survivors deal with information and communication and/or the lack of them. The way the professional-patient bond is constructed, how the communication is established and how information is transmitted, besides other factors, influence the posture of the patient in relation to his disease. In the Brazilian context, patients will follow what the doctor recommends; a majority of patients do not have enough discernment or knowledge to discuss and evaluate together with the medical team which is the best treatment to follow, for example. In other words, the low educational level of the patients that are attended by the unified health system impairs the reaction of the patient confronted with the disease due to the cascade effect. By this, I mean that the patient and the professional need to communicate and exchange information at any moment along the cancer continuum; at the same time, the pathology is “new” in the life context of that patient and he or she doesn’t understand the language used and, many times, the doctors do not explain it in another manner. To sum up, the survivors continue not understanding. Ashamed of asking questions, they simply obey and do what they are asked to do. *I just did what the doctors asked. I don’t know why I had to do that* (B). 

Continuing the idea of the cascade effect previously described, there is a moment when the patient feels cornered by the health system and simply obeying what the doctors have asked is no longer a solution. *I feel pain in my bladder all the time [starts to cry]; I don’t know what to do! The physicians don’t do anything. They know what I feel, but they always make up an excuse* (B). Here the question is how much the professionals listen to the patient and are in fact willing to help him or her to understand what is happening and solve the problem. It is noticed that, in this model, the patient is not at the center of the care; his or her opinion does not matter and what dominates is the biomedical model of treating something punctually, while what is beyond treatment does not deserve attention. These negative experiences may arise along two paths: either the patient abandons the monitoring process, which many Brazilian participants have already expressed they are willing to do, or they try to advocate for themselves. In the Brazilian context, this last path can escalate into a pattern seen among the survivors; many of them get desperate and give up on returning to the appointments and/or when there are no other options left, they seek help from other professionals, who can help them to face this necessity. It is noteworthy that the nurses can act as the patients’ advocate, which is a new attribute to the profession and deserves more attention from researchers and professionals in the field.

The Canadian participants of this study have shown to be good defenders of their own bodies and, in their discourse, they show enough knowledge about the disease to be very present in the decision taking processes. *If I have a radical prostatectomy, what are the potential outcomes? If cancer reoccurred, what would be my treatment options at that point in time? With Brachia therapy what are the, what are the outcomes with Brachia therapy? And if cancer reoccurred, what are my potential treatments with Brachia therapy? And I made the decision to have a radical prostatectomy based on more what the follow up treatments could be rather than so much what the present things were* (C). This posture may not, however, represent all Canadians; the fact of presenting self-advocacy is also related to a personality style and, thus, it is not only attached to the patient, but also extended to the professionals and institutions responsible for the health organization of the country and disease control. An example is the National Coalition for Cancer Survivorship in the United States, which has as its mission -- to advocate for quality care for people with all cancers. 

## Discussion

The findings from this study indicate that the issues raised by the participants are not different from what can be evidenced in the literature^5^ ^,^ ^9^ ^,^ ^18^ ^-^ ^20^. The interesting point is that the problems surfaced here can be similar between both countries. The difference is in how two countries, economically and culturally different, deal with the problems though, and how they manage ways of solving them, turning theory into practice, seeking quality of service, and treating the multidisciplinary team in its interactions, along with the needs of survivors, as the central point of the entire process.

It is known that, in both countries, the number of cancer survivors is growing and, although there are no statistical data on the numbers of survivors in Brazil, this country converges to the same reality as that of developed countries. Thus, sooner or later, the needs of these patients will be the same. The participants in this study have lived between four months through more than ten years following the completion of their primary treatment. Even so, they are able to report problems their health system has not yet overcome. Communication is the cornerstone of the cancer survivorship care plan, so it deserves attention. It is noteworthy that important communication is present throughout the continuum of cancer, and along the patient journey there will be needs, doubts and specific questions about the moment lived. Further studies in the area are needed, mainly in Brazil, to help identify the specific needs and challenges of each step in the cancer process, especially after the end of treatment, and to facilitate the construction of a care plan based on the socio-cultural context of each region.

In two Canadian studies, the authors evidenced that, after the conclusion of the primary treatment and as from the moment of transition to start the follow-up, the patients have seen in themselves a lot of emotions, such as confusion, insecurity, vulnerability, loss, abandonment; they feel dismissed and set adrift and often dismayed by how little attention was paid to informing them about what to expect in this phase of their cancer journey^9^. In other words, to enter into the survivorship universe typically became confusing and emotionally disturbing, instead of the sense of relief and normalcy most had expected^5^. Many had to adjust to new responsibilities associated with work and family post-treatment and were concurrently coping with feelings of loss or/and abandonment arising from an abrupt separation from the feeling of security that regular contact with experts afforded. What helped many during this emotionally complex period was assistance to contextualize their new state of “normal”, including anticipation and management of these dependencies that had arisen during the active treatment phase^5^.

Despite widespread acknowledgement of the importance of communication for both quality of life and cancer outcomes in cancer care, the evidence continues to confirm that significant numbers of patients cannot access suitable communication^5^. Moreover, there are still barriers that hinder the exchange of information and effective communication. These barriers are multiple and interrelated and linked to both the issuers and receivers of the communication. The life-threatening nature of the illness, the physical and psychological suffering of cancer patients^11^ ^,^ ^21^ ^)^ and the responsibility of conveying complex health information to the patients while also managing their emotions^10^ besides insufficient training, low self-efficacy and poor emotional regulation^22^, are some of the challenges that affect communication in the professional and patient dyad in the context of cancer. Authors who have been working in this field have found over a decade that cancer communication is so complex and difficult that effectiveness seems improbable^5^. Nevertheless, from the patients’ perspective, it is apparent that patients expect individualized approaches to communication and that involves awareness of their individually distinct contexts. They prefer that relevant communications occur within the context of the human connections associated with effective healthcare relationships^5^.

In one study^22^, the authors realized that, although the majority of care models that were developed and tested in Western cultures kept the focus on individuality, their findings noted the importance of developing culturally appropriate care and education for the patients in their context. Culture is a symbolic and public system focused on the individual^23^. Therefore, culture is learned, shared and standardized, and its constituent elements are values, symbols, rules and practices. Thus, communication that takes into account the cultural issues might improve access to healthcare and quality of healthcare, and reduce and/or eliminate health disparities^10^ ^-^ ^11^. Internationally, there are already studies^10^ ^,^ ^11^ ^,^ ^24^ that address the issue of culture associated with communication and treat this junction as a requirement for health professionals working in the cancer setting. Indeed, it seems timely and important to delve into qualitative interpretive studies that can enhance our understanding of the issue of culture with respect to communication for health care professionals and cancer survivors.

Although not mentioned by the Brazilian participants, the nurse has an important role in the construction of good communication and, consequently, a reliable relationship. Doctors and nurses need to be clear and honest with the patients. They should spend enough time with patients to have personal, trustful relationships^18^. There are some aspects of effective communication that only apply to nurses; these include educating patients about lifestyle and health promotion, giving support, spreading good humor and cheer, serving as a go-between for doctors and patients, balancing professionalism and close relationships with patients, and checking whether patients have everything they need^18^.

This study includes perspectives for all professionals involved in health care within the cancer survivorship process, especially for nursing, in which the nurse acts as a key part in all thematic patterns outlined here. It is already known that the healthcare professional’s ability to effectively and sensitively communicate with cancer patients and their family has a huge impact on the cancer survivorship experience. That ability is honed through years of intricately weaving together theoretical knowledge, evidence derived from research, ethical reasoning, experiences and critical self-reflection about patient care experiences, in conjunction with the nurse’s own evolving capacity for therapeutic communication^25^.

## Conclusion

The results of this study complement themes in the existing literature with regard to the interaction between survivor/patient and health professional, drawing attention to various aspects of communication in the construction of this interaction, beyond the search for information and the empowering of the patients when it comes to their body and self-advocacy, seeking the best care strategies, where they can be heard, and how their opinions can be taken into consideration. This comparative study allowed us to bring to the attention of health professionals, especially nurses, regarding effective communication, the value of humanization, empathy, supporting inside and outside support groups, and giving advice. Clearly advocating for the survivor is always necessary, beyond showing the importance of self-development of these professionals and also fighting for a better quality in the health system. It can be seen that the Brazilian health system remains in a situation of enacting health policies in theory, with little evidence in practice. It is necessary to build research programs that will make explicit the optimal role of the nurse in relation to warranting cancer survivors’ quality of life, especially after the end of the treatment, when they bear the complications of the disease and, at the same time, have a strong desire to return to their “normal” social lives. 
